# Whole-exome sequencing revealed a novel *ERCC6* variant in a Vietnamese patient with Cockayne syndrome

**DOI:** 10.1038/s41439-022-00200-1

**Published:** 2022-06-06

**Authors:** Nguyen Thuy Duong, Nguyen Phuong Anh, Nguyen Duy Bac, Le Bach Quang, Noriko Miyake, Nong Van Hai, Naomichi Matsumoto

**Affiliations:** 1 Institute of Genome Research, Vietnam Academy of Science and Technology, Hanoi, Vietnam; 2 Vietnam Military Medical University, Hanoi, Vietnam; 3 Yokohama City University Graduate School of Medicine, Yokohama, Japan; 4 National Research Institute, National Center for Global Health and Medicine, Tokyo, Japan

**Keywords:** Mutation, Neurodegenerative diseases

## Abstract

We describe a case of Cockayne syndrome without photosensitivity in a Vietnamese family. This lack of photosensitivity prevented the establishment of a confirmed medical clinical diagnosis for 16 years. Whole-exome sequencing (WES) identified a novel missense variant combined with a known nonsense variant in the *ERCC6* gene, NM_000124.4: c.[2839C>T;2936A>G], p.[R947*;K979R]. This case emphasizes the importance of WES in investigating the etiology of a disease when patients do not present the complete clinical phenotypes of Cockayne syndrome.

Cockayne syndrome (CS) is a rare autosomal recessive genetic disorder that is characterized by growth failure, microcephaly, and premature aging. The signs and symptoms of this disease usually appear during childhood and worsen over time. Patients with CS are very sensitive to sunlight. In some cases, even short exposure to the sun can cause sunburn or blisters. Based on the age of onset and the spectrum of severity, CS can be divided into three subtypes: the classic/moderate form (type I), the severe/early-onset form (type II), and the mild/late-onset form (type III)^1^. CS type I patients usually develop symptoms in the first two years and have an average lifespan of 16 years^2,3^. Patients with CS type II present more severe symptoms at birth and rarely survive beyond the age of five^2,3^. CS type III patients have normal development in the first two years and then express typical clinical manifestations^4,5^. Patients with this type can live to be over 30 years old^2,3,6^.

Recessive variants in either the *ERCC6* (MIM #133540; CS-B) or *ERCC8* (MIM #216400) gene can cause Cockayne syndrome. *ERCC6* encodes the DNA excision repair protein ERCC6 (CSB). CSB is a 1493-amino-acid protein that contains seven helicase-like ATPase motifs. The CSB protein belongs to the SWI2/SNF2 helicase family^7^ and is involved in chromosomal remodeling, transcription regulation, and DNA repair^8,9^. *ERCC8* encodes the DNA excision repair protein ERCC8 (CSA). CSA is a 396-amino-acid protein that comprises seven WD (tryptophan-aspartic acid dipeptide) domains^5,10^. CS proteins play an important role in the TC-NER (transcription-coupled nucleotide excision repair) process, and TC-NER-deficient CS cells demonstrate increased sensitivity to UV irradiation^11^. Therefore, pathogenic variants in CSA and CSB result in TC-NER deficiency and cause various hereditary diseases, including CS^12^. In total, *ERCC6* mutations account for ~70% of cases, and *ERCC8* mutations account for ~30%^5,13^.

We identified a novel variant, c.2936A>G p.K979R, and one known variant, c.2839C>T, p.R947*, in the *ERCC6* gene in a Vietnamese patient with CS using whole-exome sequencing.

Blood samples were collected from all family members after obtaining informed consent from the parents. This study was approved by the Institutional Review Board of the Institute of Genome Research, Vietnam Academy of Science and Technology, and the Yokohama City University Faculty of Medicine.

Genomic DNA was extracted and purified from peripheral blood using the GeneJET Whole Blood Genomic DNA Purification Mini Kit (ThermoFisher Scientific, USA) following the manufacturer’s protocol. Whole-exome sequencing (WES) was performed on the proband (II-2). Genomic DNA was partitioned using the SureSelectXT Human All Exon 50 Mb, v5 libraries (Agilent Technologies, Santa Clara, CA, USA) on a HiSeq 2500 platform (Illumina) with 101 bp paired-end reads. Short reads were mapped onto a human reference genome (UCSC hg19) using Novoalign (http://www.novocraft.com/products/novoalign/). PCR duplications were filtered out by Picard version 2.18.7 (http://broadinstitute.github.io/picard/). Variant calling was performed following Genome Analysis Toolkit Best Practices (https://www.broadinstitute.org/gatk/index.php)^14^.

The proband (II-2, Fig. 1) was 16 years old at the time of our study. She was born at full-term with a weight of 3.2 kg and looked normal at birth. At the age of one, she had fever and diarrhea for a month. From the age of two, she suffered from slow growth. She ate very little. At the age of three, her weight and height were 8 kg (13.9 ± 1.8) and 70 cm (95.1 ± 3.85), respectively. She was not able to speak whole sentences. At 10 years, her face showed signs of premature aging, and she suffered hair loss (Fig. 1A, B). She was unable to stand up and was completely paralyzed. She moved around with her two arms and had urinary incontinence. Two years later, the proband’s body stopped growing, and she did not gain weight. At the age of 17, the proband died of severe pneumonia.

**Fig. 1:**
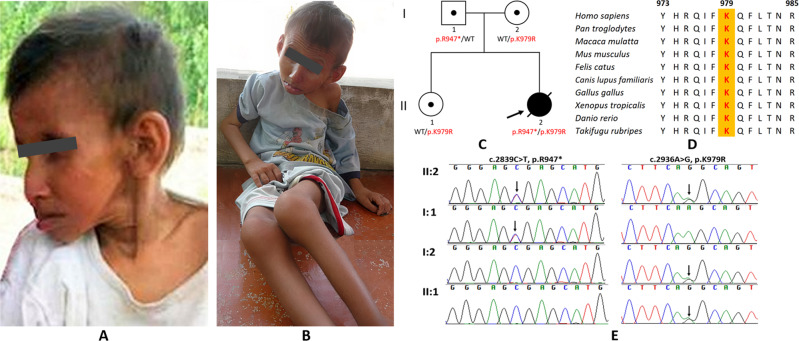
Proband at the age of 16 and the pedigree analysis of the family in the study. Sitting posture of II-2 from sidewise (**A**) and front (**B**). The family pedigree of the proband (**C**). Multiple sequence alignment at amino acid position p.K979 among different species (**D**). Sanger sequencing results of the proband, the proband’s healthy sister, and the proband’s healthy parents (**E**).

Using WES, two variants (p.R947*; p.K979R) on the *ERCC6* gene were identified in the proband. The Sanger sequencing results showed that the c.2839C>T, p.R947* variant was inherited from the patient’s father, and the c.2936A>G, p.K979R variant was inherited from her mother. The patient’s sister (II-1) also carried p.K979R (Fig. 1C). The amino acid at position 979 (Lys979) of the CSB protein is conserved among different species. The p.K979R variant was predicted to be damaging using ten in silico prediction tools (SIFT, Polyphen-2, MutationTaster, FATHMM, MutationAssessor, MutPred 2, Meta-SNP, PROVEAN, SNAP, and PANTHER).

The proband was initially diagnosed with progeria syndrome, and she was screened for pathogenic variants in *LMNA* and *ZMPSTE24*. However, the Sanger sequencing results showed no pathogenic changes in these two genes. Therefore, we performed WES on the proband and screened for all pathogenic variants in all genes associated with a group of aging-related diseases. WES revealed two variants in the *ERCC6* gene, NM_000124.4: c.2839C>T, p.R947* and c.2936A>G, p.K979R, in the patient. Segregation analysis showed that c.2839C>T, p.R947* was found on the paternal allele, and c.2936A>G, p.K979R was found on the maternal allele.

The c.2839C>T, p.R947* variant was previously reported in some studies^15,16^. However, this variant has not been reported to cause disease in the African American patient with Cockayne syndrome type II^15^. This patient had a missense variant, p.P1095R, in addition to another heterozygous variant, p.K506Nfs*37, that caused a premature termination codon 404 amino acids upstream of the c.2839C>T, p.R947* variant on the same allele. The mutated residue p.R947 is positioned in the SNF2/ATPase domain. This domain extends from amino acids 510 to 960 and consists of seven ATPase motifs: I, Ia, II, III, IV, V, and VI^17^. This domain is extremely conserved in the SWI2/SNF2 family^18^ and is essential for the functioning of the CSB protein^19,20^. Its function strengthens the association between CSB and chromatin by changing the positions of the nucleosomes^21^. Through this action, CSB helps repair DNA damage by promoting other proteins (e.g., CSA and NER factors) that affect the location at which RNA pol II stalls^22^. Therefore, mutations and amino acid substitutions in this domain might impair cell survival, reduce the recovery of RNA synthesis and inhibit the repair of DNA after UV exposure^5^.

The c.2936A>G variant leads to the conversion of the amino acid lysine to arginine at position 979, which is located in a highly conserved region of the CSB protein (Fig. 1D). This indicates the functional importance of this protein. The p.K979 variant is positioned between the ATPase region (residues 510–960) and the nuclear localization signal (residues 1038–1055)^5^. Two missense variants were found in this region; however, they exhibit a broad spectrum of interfamily clinical manifestations. The p.R975W variant was found in combination with a nonsense mutation in a Chinese patient with CS^23^, and the p.L987P variant was found together with another splicing variant in a French patient with cerebro-oculo-facio-skeletal syndrome (COFS)^24^. The CS-prominent clinical feature of photosensitivity was absent in our patient and the French patient but was present in the Chinese patient. Moreover, all the common phenotypes presenting in our patient were similar to those in the patient with COFS, which is an allelic disorder of CS. However, these phenotypes were less severe in these two patients than in the Chinese patient. These findings suggest that there may be factors other than the location of the pathogenic variants that influence the type and severity of the clinical features of CS.

In conclusion, we report two pathogenic variants, including the novel c.2936A>G p.K979R variant and the known c.2839C>T, p.R947* variant, in the *ERCC6* gene that were inherited in an autosomal recessive pattern. The segregation of these variants was confirmed in the family using Sanger sequencing (Fig. 1E). The findings of our study enrich the genetic spectrum of Cockayne syndrome and highlight the value of whole-exome sequencing in identifying pathogenic variants of genetic diseases, especially diseases with overlapping symptoms.

## HGV database

The relevant data from this Data Report are hosted at the Human Genome Variation Database at 10.6084/m9.figshare.hgv.3203.
